# Clubroot-Induced Changes in the Root and Rhizosphere Microbiome of Susceptible and Resistant Canola

**DOI:** 10.3390/plants13131880

**Published:** 2024-07-08

**Authors:** Jorge Cordero-Elvia, Leonardo Galindo-González, Rudolph Fredua-Agyeman, Sheau-Fang Hwang, Stephen E. Strelkov

**Affiliations:** 1 Department of Agricultural, Food and Nutritional Sciences, University of Alberta, Edmonton, AB T6G2P5, Canada; jcordero@ualberta.ca (J.C.-E.); leonardo.galindogonzalez@inspection.gc.ca (L.G.-G.); freduaag@ualberta.ca (R.F.-A.); sh20@ualberta.ca (S.-F.H.); 2 Ottawa Plant Laboratory, Science Branch, Canadian Food Inspection Agency, 3851 Fallowfield Road, Ottawa, ON K2H8P9, Canada

**Keywords:** biocontrol, clubroot resistance, *Plasmodiophora brassicae*, virulence, root microorganisms

## Abstract

Clubroot is a soilborne disease of canola (*Brassica napus*) and other crucifers caused by the obligate parasite *Plasmodiophora brassicae*. In western Canada, clubroot is usually managed by planting-resistant cultivars, but the emergence of resistance-breaking pathotypes of *P. brassicae* represents a major threat to sustainable canola production. The rhizosphere and root contain beneficial microorganisms that can improve plant health. In this study, we evaluated the effect of two *P. brassicae* isolates (termed A and B) with different levels of virulence on the root and rhizosphere microbiomes of clubroot-resistant and clubroot-susceptible canola. Additionally, potential biocontrol microorganisms were identified based on taxa antagonistic to clubroot. Although both *P. brassicae* isolates were classified as pathotype 3A, isolate A caused a higher disease severity index in the resistant canola genotype compared with isolate B. Metabarcoding analysis indicated a shift in the bacterial and fungal communities in response to inoculation with either field isolate. Root endophytic bacterial and fungal communities responded to changes in inoculation, isolate type, sampling time, and canola genotype. In contrast, fungal communities associated with the rhizosphere exhibited significant differences between sampling times, while bacterial communities associated with the rhizosphere exhibited low variability.

## 1. Introduction

Clubroot is a soilborne disease of the Brassicaceae family caused by the obligate parasite *Plasmodiophora brassicae* Woronin. The disease can cause significant losses in canola (syn. oilseed rape; *Brassica napus* L.) production in Canada. Susceptible plants infected by *P. brassicae* develop root galls, which decrease their capacity for water and nutrient uptake. This can lead to wilting and death of the plant when symptoms are severe [1]. The clubroot disease cycle consists of three phases: survival in soil, primary infection of the root hairs, and secondary infection of the cortex tissue [2]. The primary infection phase mainly occurs from 0 to 7 days after inoculation (dai), whereas the secondary infection phase starts from 7 dai and lasts up to 24 dai [3]. Clubroot has been spreading rapidly through the Canadian prairies and has become a significant challenge for canola producers [4,5]. The increasing occurrence of *P. brassicae* is associated with its production of large numbers of long-lived resting spores, which can survive in the soil for many years and serve as the inoculum for infecting subsequent crops [6,7]. Agronomic management practices for clubroot control include crop rotation, sanitization of field equipment, and the application of fungicides and soil amendments [8]. Unfortunately, many of these strategies are not practical or cost-effective [9], and the most widely used approach for clubroot management has been the planting of clubroot-resistant canola cultivars [10,11,12]. In recent years, however, new pathotypes of *P. brassicae* that are highly virulent on resistant canola have emerged; among these, pathotype 3A is predominant in western Canada [5,13]. 

The implementation of an integrated approach for clubroot mitigation must consider protective agronomic practices, along with improved clubroot resistance and soil health. In this context, the rhizospheric and endophytic microbiomes associated with crops include beneficial microorganisms that can reduce pathogen proliferation via several mechanisms. These mechanisms include the synthesis of inhibitory allelochemicals such as antibiotics, hydrogen cyanide, volatile compounds, siderophores, and antifungal metabolites [14,15]. Other mechanisms can reduce pathogen infection indirectly by inducing the activation of plant defense systems that inhibit the activity of a wide range of phytopathogens [3,16,17,18,19,20,21]. These microbial communities can also alter soil quality, improve plant nutrient uptake, and stimulate plant growth [18,19,20]. In the case of clubroot, previous studies showed that several bacterial and fungal species have antagonistic effects against *P. brassicae*, including species of *Bacillus*, *Lysobacter*, *Streptomyces*, and *Zhihengliuella* [22,23,24,25,26,27]. Similarly, some fungal species such as *Trichoderma* and *Gliocladium* are reported as clubroot antagonists [22,28,29]. Some mechanisms used by these microbes to control clubroot involve the production of hydrolytic enzymes, the synthesis of diverse secondary metabolites, and the activation of plant defense systems [28,30,31].

Microbiome studies of the rhizosphere and root endosphere of crops have emphasized the high complexity and variability of microbial communities [32,33]. A recent investigation assessing the effect of the soil microbiome on *P. brassicae* during pathogenesis indicated that soil microbial diversity had an influence on clubroot development [34]. However, the effect of diversity on symptom severity and the pathogen varied among canola genotypes. Similarly, another study showed that the microbial diversity and relative abundance associated with clubroot-asymptomatic *B. napus* roots were higher than in symptomatic roots. Asymptomatic roots harbored several microbes with biological control and plant growth promotion functions that may be related to the absence of clubroot symptoms [35]. Such findings suggest that pathogen suppression is controlled by microbial communities rather than individual species. In addition, several factors, such as plant species and/or cultivars and plant growth stage, are known to influence the interactions that occur between host crops and bacterial endophytes [36]. Therefore, understanding shifts in microbiome composition in response to changes in clubroot incidence, canola genotype, and growth stage can improve our knowledge and aid in generating biocontrol strategies against clubroot. In the current study, the effect of two *P. brassicae* isolates (pathotype 3A), exhibiting different levels of virulence, on the root and rhizosphere microbiomes of clubroot-resistant and clubroot-susceptible canola was assessed. Additionally, potential biocontrol microorganisms were identified based on taxa antagonistic to clubroot. Our results indicate a shift in the community profiles of the bacteria and fungi associated with the roots, as well as fungi associated with the rhizosphere soil of the studied canola lines, in response to changes in inoculation, isolate, and sampling time. In contrast, bacterial communities associated with the rhizosphere exhibited lower variability. Furthermore, an increase in the relative abundance of several bacterial species was detected in resistant inoculated plants, suggesting that these microorganisms have potential utility as biocontrol agents against clubroot.

## 2. Results

### 2.1. Clubroot Symptoms, Disease Index, and Pathotyping

Plants harvested at 7 days after inoculation (dai) showed similar disease symptoms to both clubroot isolates (Figures S1–S4). Both canola lines did not exhibit differences between control and inoculated plants at 7 dai. At 21 and 35 dai, susceptible inoculated plants were smaller than the other treatments, and most exhibited root galling with both isolates. In contrast, the resistant canola lines showed a differential response to both clubroot isolates at 21 and 35 dai. The resistant line was smaller than the controls when inoculated with isolate A, but when inoculated with isolate B, the resistant plants exhibited a similar size to the controls (Figures S1–S4). 

**Figure 1:**
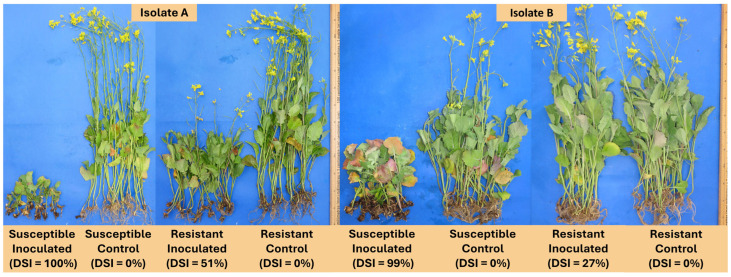
Clubroot disease severity index (DSI) on clubroot-resistant and clubroot-susceptible canola lines, 42 days after inoculation with two isolates (A and B) of *Plasmodiophora brassicae* (pathotype 3A). Non-inoculated plants served as controls. Canola plants were cultivated in the greenhouse using a completed randomized design with 3 replicates. Inoculation with either isolate produced high DSI values on the susceptible canola plants. Resistant canola inoculated with isolate A exhibited higher DSI values compared to those inoculated with isolate B.

The disease severity index (DSI) results at 42 dai indicated a significant effect of clubroot inoculation on the susceptible canola line (DSI = 100%) compared to the uninoculated controls (DSI = 0%) for both clubroot isolates tested (Figure 1). The resistant line also exhibited symptoms of clubroot infection in response to isolate A, but the DSI values were lower (51%) compared with the susceptible line (100%). These results indicate that the canola line 12PH0277 was only moderately resistant to isolate A. The DSI of the resistant line was lower (27%) when inoculated with isolate B compared to isolate A (Figure 1), suggesting that the isolate virulence influenced the reaction of the resistant canola line. Despite the different levels of virulence, both isolates were classified as pathotype 3A on the Canadian Clubroot Differential (CCD) set [5], pathotype 3 on the system of Williams [37], and pathotype P_2_ according to the system of Some et al. [38]. The reactions of most differential hosts to inoculation were very similar for both isolates, with only the rutabaga (*B. napus* var. napobrassica L.) hosts (ECD 10 and ‘Laurentian’) developing more severe symptoms in response to isolate A. Nevertheless, these two hosts were still susceptible to isolate B (Figure S5).

### 2.2. Effect of P. brassicae Inoculation on Fungal Communities Associated with Canola

**Figure 2:**
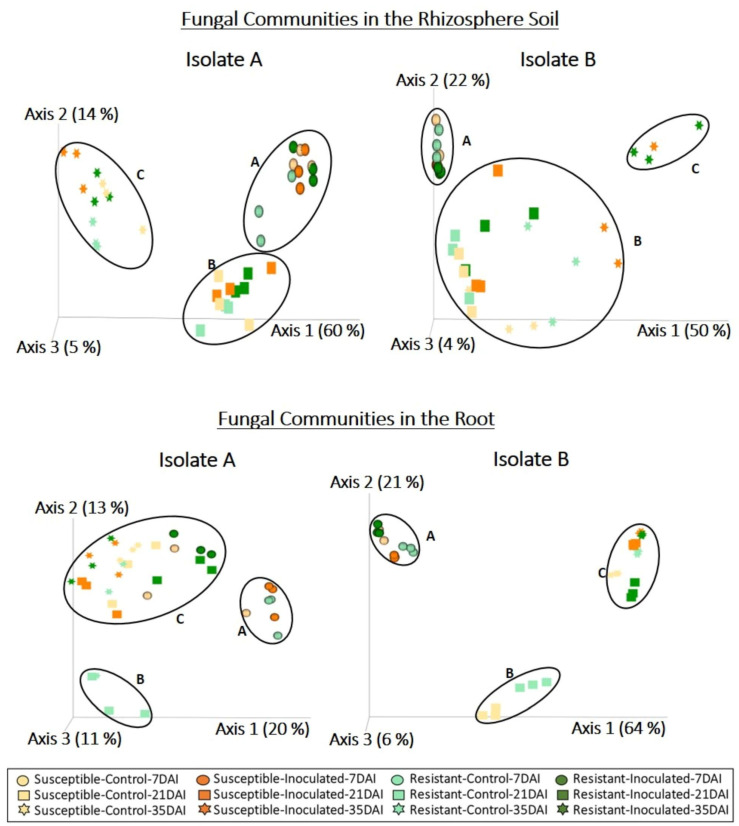
Principal coordinates analysis of fungal communities associated with rhizosphere soil and root samples. Three replicates from clubroot-resistant and -susceptible canola lines were sampled at 7, 21, and 35 days after inoculation (dai) with two isolates (A and B) of *Plasmodiophora brassicae* (pathotype 3A). Non-inoculated plants served as controls. Fungal profiles were obtained from the sequence analysis of the ITS region using QIIME2. Fungal communities associated with the rhizosphere and root interior mainly differed in sampling times. Inoculation treatment and *P. brassicae* isolate also influenced the clustering of fungal communities in the rhizosphere and root samples (clusters A, B and C).

DNA extracted from the rhizosphere soil and roots of clubroot-susceptible and resistant canola lines was used to amplify the ITS region, which provides high resolution for identifying fungal genera and species. Analysis of ITS sequences derived from the rhizosphere soil resulted in 1,146,578 quality-filtered reads for the dataset with isolate A and 1,041,744 reads for the dataset with isolate B. Taxonomic classification of sequence reads derived from the rhizosphere soil detected 423 amplicon sequence variants (ASVs) following isolate A inoculation and 353 ASVs following isolate B inoculation. Principal coordinates analysis based on the relative abundance of detected ASVs indicated a shift in the fungal communities associated with the rhizosphere of both canola lines in response to sampling time with both isolates (Figure 2, Table 1). Principal coordinates ordination revealed the presence of three clusters corresponding to samples collected at 7 dai (cluster A), 21 dai (cluster B), and 35 dai (cluster C) in response to inoculation with isolate A (Figure 2). Fungal profiles of the rhizosphere of canola inoculated with isolate B (Figure 2) also included a cluster of samples collected at 7 dai (cluster A). However, cluster B comprised samples collected at 21 dai and the controls of 35 dai, whereas cluster C included only inoculated samples collected at 35 dai. Permanova results confirm a significant effect of sampling time in the fungal communities associated with the rhizosphere for both isolates, but for isolate B, a significant interaction was seen for inoculation and time (Table 1).

For the root samples, analysis of ITS sequences resulted in 186,654 and 565,016 quality-filtered reads for the datasets corresponding to isolates A and B, respectively. Classification of sequence reads derived from the roots detected 101 ASVs following inoculation with isolate A and 142 ASVs following inoculation with isolate B. Fungal communities associated with the roots formed a cluster that included most of the samples collected at 7 dai (cluster A) for both isolates (Figure 2). However, Cluster B included the resistant canola control collected at 21 dai for both isolates, as well as the susceptible control for isolate B. Cluster C encompassed all remaining samples collected at 21 and 35 dai. Permanova analysis showed a significant interaction of sampling time and inoculation in the fungal communities associated with roots for both isolates (Table 1).

**Figure 3:**
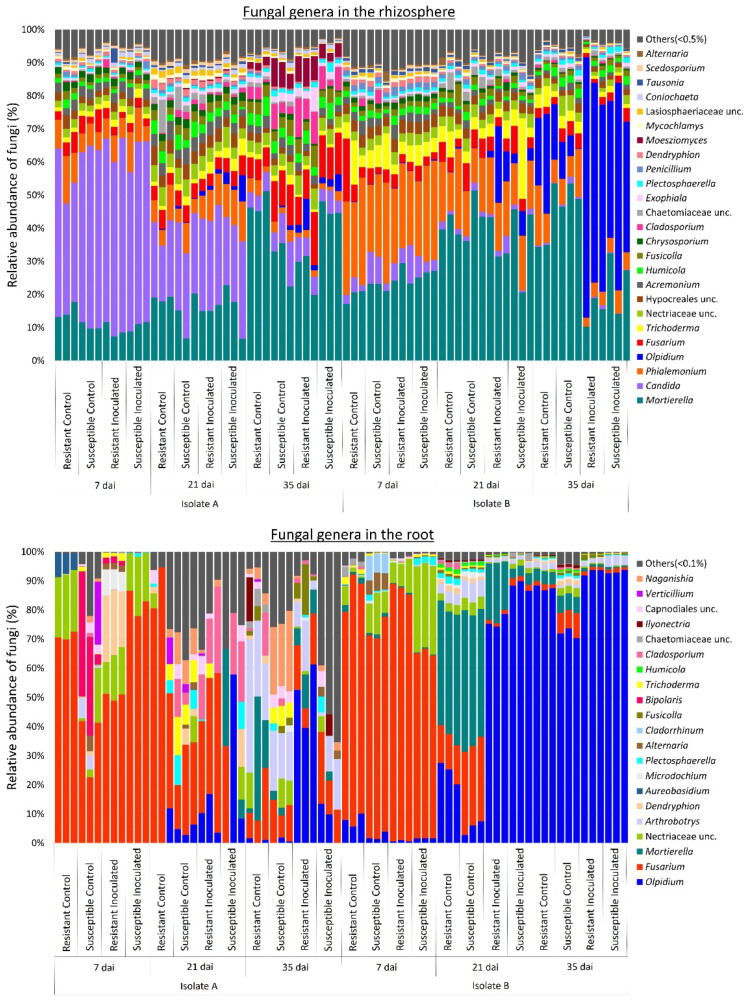
Relative abundance of fungal genera associated with rhizosphere soil and root samples. Three replicates of clubroot-resistant and -susceptible canola lines were sampled at 7, 21, and 35 days after inoculation (dai) with two isolates (A and B) of *Plasmodiophora brassicae* (pathotype 3A). Non-inoculated plants served as controls. Fungal taxonomy and abundance were obtained from the sequence analysis of the ITS region using QIIME2. The relative abundance of fungal genera associated with the rhizosphere and roots varied in response to sampling times, canola lines, inoculation treatment, and *P. brassicae* isolate.

Given the observed changes in fungal communities associated with the rhizosphere and roots of canola lines across different *P. brassicae* isolates and sampling times, we pooled the taxonomic data (Figure 3). This was done by calculating the average relative abundance (%) of these genera for each factor (i.e., inoculant type and sampling time) to assess their respective effects on fungal communities. In the rhizosphere, the genera *Mortierella* (14%), *Candida* (9%), *Phialemonium* (3%), and *Fusarium* (3%) were the most abundant in samples corresponding to isolate A, whereas for isolate B, the most abundant genera were *Mortierella* (27%), *Olpidium* (13%), *Phialemonium* (8%), *Trichoderma* (3%), and *Fusarium* (3%) (Figure 3). The genus *Candida* was predominant in samples with isolate A at 7 and 21 dai, whereas *Mortierella*, *Fusarium*, *Cladosporium*, and *Moesziomyces* were more abundant at 35 dai. In contrast, in samples with isolate B, *Phialemonium* was more abundant at 7 and 21 dai, whereas *Olpidium* was predominant at 35 dai in inoculated plants for both canola lines. 

In the roots, *Fusarium* (37%), *Olpidium* (8%), unclassified Nectriaceae (7%), and *Arthrobotrys* (6%) were the most abundant genera in samples corresponding to isolate A, whereas *Olpidium* (56%), *Fusarium* (20%), *Mortierella* (12%), and unclassified Nectriaceae (4%) were more abundant in samples corresponding to isolate B (Figure 3). For both isolates, the relative abundance of *Fusarium* and unclassified Nectriaceae was higher at 7 and 21 dai. At 21 and 35 dai, the genus *Olpidium* exhibited higher abundance, particularly in samples with isolate B, where it was the predominant fungus during those sampling times. Conversely, for isolate A, the abundance of *Cladosporium* and *Arthrobotrys* was higher at 21 and 35 dai, respectively. Additionally, *Naganishia* showed higher abundance in control samples during those sampling times.

**Figure 4:**
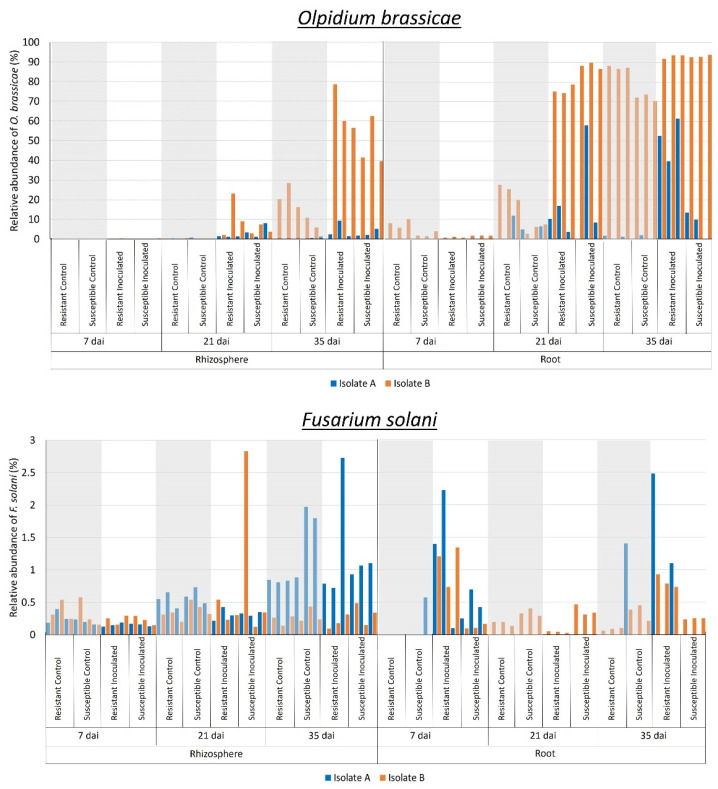
Relative abundance of *Olpidium brassicae* and *Fusarium solani* associated with rhizosphere soil and root samples. Three replicates of clubroot-resistant and -susceptible canola lines were sampled at 7, 21, and 35 days after inoculation (dai) with two isolates (A and B) of *Plasmodiophora brassicae* (pathotype 3A). Non-inoculated plants served as controls (gray area). Fungal taxonomy and abundance were obtained from the sequence analysis of the ITS region using QIIME2. The relative abundance of *O. brassicae* increased in the root endosphere of samples inoculated with either *P. brassicae* isolate at 21 and 35 dai. *F. solani* was more abundant in the rhizosphere at 35 and 21 dai with isolates A and B, respectively, whereas in the root, this genus was more abundant in inoculated samples collected at 7 and 35 dai for both isolates.

Differential abundance analysis of fungal communities at the species level between control and inoculated canola plants indicated an increase in the relative abundance of *Olpidium brassicae* in samples inoculated with *P. brassicae* at 21 and 35 dai. Specifically, this increase was observed in the rhizosphere (W > 1000) for isolate B and in the root interior (W > 200) for both isolates (Figure 4). Similarly, *Fusarium solani* was more abundant in the rhizosphere (W > 1000) of inoculated plants at 35 and 21 dai for isolates A and B, respectively (Figure 4). In the root (W > 200), this fungus was more abundant in samples collected at 7 and 35 dai for both isolates (Figure 4). Fungal profiles at genus level (Figure 3) confirmed that *Olpidium* was more abundant in inoculated samples collected at 21 and 35 dai in the rhizosphere for isolate B, as well as in the root interior for both isolates. The abundance of *Fusarium* was also higher in the roots of inoculated samples collected at 21 and 35 dai for isolate B (Figure 3).

### 2.3. Effect of P. brassicae Inoculation on Bacterial Communities Associated with Canola

**Figure 5:**
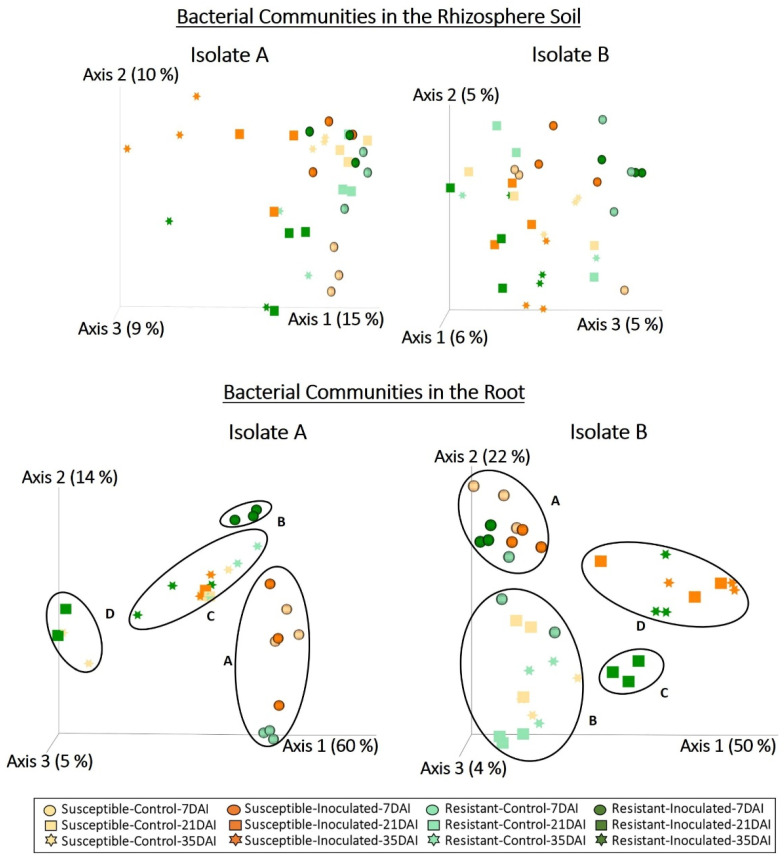
Principal coordinates analysis of bacterial communities associated with rhizosphere soil and root samples. Three replicates of clubroot-resistant and -susceptible canola lines were sampled at 7, 21, and 35 days after inoculation (dai) with two isolates (A and B) of *Plasmodiophora brassicae* (pathotype 3A). Non-inoculated plants served as controls. Bacterial profiles were obtained from the sequence analysis of the 16S rRNA region using QIIME2. Bacterial communities associated with the rhizosphere exhibited no clustering in response to the treatments. Inoculation treatment, isolate type, sampling time, and canola lines influenced the clustering of bacterial communities in the root (clusters A, B, C and D).

Bacterial communities associated with rhizosphere soil and roots of clubroot-resistant and -susceptible canola lines were analyzed by high throughput sequencing of 16S rRNA amplicons. Analysis of the 16S rRNA sequences derived from the rhizosphere resulted in 1,554,798 quality-filtered reads for the dataset corresponding to isolate A and 1,722,644 reads for the dataset corresponding to isolate B. Taxonomic classification of sequence reads derived from the rhizosphere soil detected 2235 ASVs following inoculation with isolate A and 1569 ASVs following inoculation with isolate B. Bacterial profiles associated with the rhizosphere did not cluster in response to any of the factors (Figure 5). However, significant interactions between canola line and time, as well as inoculation and time, were detected by Permanova (Table 1). 

For the root samples, analysis of the 16S rRNA sequences resulted in 322,091 quality-filtered reads for the dataset corresponding to isolate A and 563,692 reads for the dataset corresponding to isolate B. Classification of sequence reads derived from the roots detected 654 ASVs following inoculation with isolate A and 1038 ASVs following inoculation with isolate B. In contrast to the rhizosphere soil profiles, bacterial communities associated with the roots revealed that most samples collected at 7 dai grouped together (cluster A) for both isolates and canola lines (Figure 5). However, for isolate A, samples from resistant plants collected at 7 dai clustered separately (cluster B). For isolate B, most control samples collected at 21 and 35 dai clustered together (cluster B). Cluster C comprised resistant inoculated samples at 21 dai for both isolates, whereas cluster D included the remaining samples collected at 21 and 35 dai. Permanova showed significant interactions between sampling time and inoculation, as well as between canola line and inoculation, in the bacterial communities associated with roots for both isolates (Table 1).

**Figure 6:**
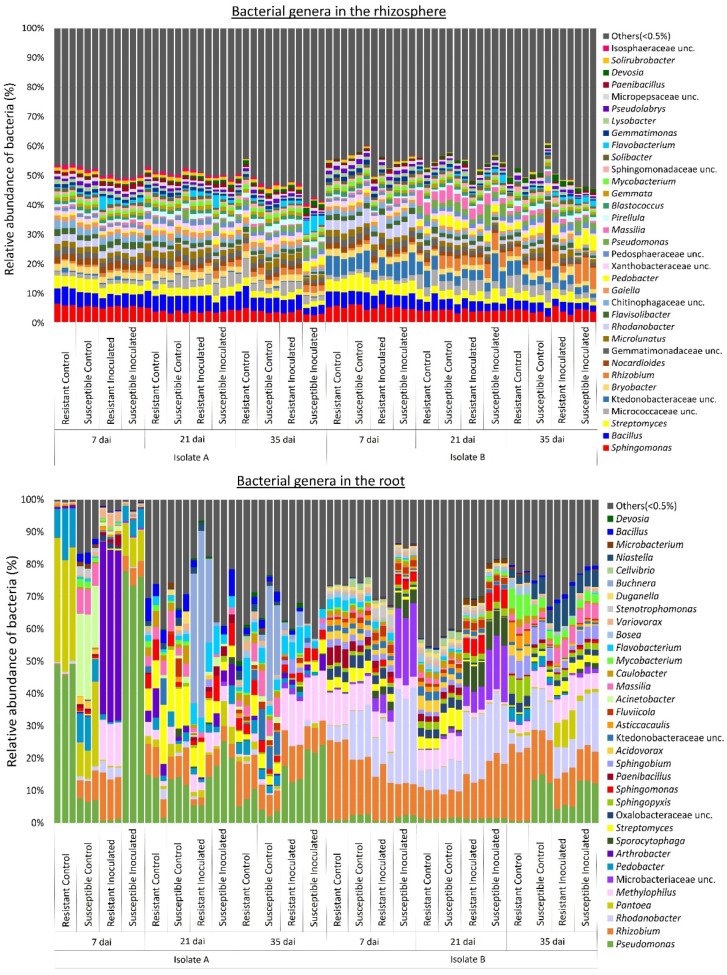
Relative abundance of bacterial genera associated with rhizosphere soil and root samples. Three replicates of clubroot-resistant and -susceptible canola lines were sampled at 7, 21, and 35 days after inoculation (dai) with two isolates (A and B) of *Plasmodiophora brassicae* (pathotype 3A). Non-inoculated plants served as controls. Bacterial taxonomy and abundance were obtained from the sequence analysis of the 16S rRNA region using QIIME2. The relative abundance of bacterial genera associated with the roots varied in response to sampling times, canola lines, inoculation treatment, and *P. brassicae* isolate. Bacterial profiles in the rhizosphere indicated an even distribution of the relative abundance among samples.

At the genus level, bacterial profiles in the rhizosphere indicated an even distribution of the relative abundance among samples (Figure 6). The genera *Sphingomonas* (5%), *Bacillus* (4%), and *Streptomyces* (3%) were the most abundant in the rhizosphere. Some genera, such as *Massilia*, *Nocardioides*, *Pedobacter*, *Rhizobium*, *Rhodanobacter*, unclassified Ktedonobacteraceae, and unclassified Sphingomonadaceae, were more abundant in samples with isolate B. The genus *Nocardiodes* was predominant in the rhizosphere sample of a control susceptible canola (isolate B) collected at 35 dai (Figure 6). In the roots, bacterial profiles at the genus level exhibited high variability across sampling times, canola lines, *P. brassicae* inoculation status, and isolate (Figure 6). The genera *Pseudomonas* (42%), *Pantoea* (19%), *Pedobacter* (7%), and *Arthrobacter* (7%) were more abundant in samples corresponding to isolate A, whereas *Pseudomonas* (15%), *Rhizobium* (12%), *Massilia* (6%), and *Methylophilus* (6%) were more abundant in samples corresponding to isolate B. For isolate A, the relative abundance of *Pseudomas* was higher in the resistant control and susceptible inoculated plants at 7 dai, decreasing at 21 and 35 dai. However, for isolate B, the relative abundance of *Pseudomonas* remained consistent across all sampling times. Most root samples collected at 7 dai corresponding to isolate A inoculation exhibited a high abundance of *Pantoea*, except for the resistant inoculated canola. In addition, root samples of resistant canola inoculated with isolate A also had a high abundance of *Arthrobacter* at 7 dai, whereas *Acinetobacter* was more abundant in susceptible canola controls at the same sampling time. In contrast, samples corresponding to isolate B collected at 7 dai exhibited a high abundance of *Mycobacterium*, while *Pedobacter* was predominant in susceptible controls and inoculated plants. The abundance of *Flavobacterium* in samples corresponding to isolate A was lower at 7 dai and increased at 21 and 35 dai. *Buchnera* was predominant at 21 dai in resistant plants inoculated with isolate A and at 35 dai in susceptible controls. The genus *Methylophilus* exhibited a higher abundance in both susceptible and resistant canola inoculated with either isolate and collected at 35 dai. *Methylophilus* was also predominant in resistant plants inoculated with isolate A collected at 7 dai and susceptible canola inoculated with isolate B collected at 21 dai. At 21 dai with isolate A, *Streptomyces* was abundant in controls and resistant inoculated plants, while with isolate B, *Streptomyces* was predominant in control plants collected at 35 dai. *Massilia* was abundant in most samples corresponding to isolate B, except for susceptible inoculated canola at 21 dai and both inoculated canola lines at 35 dai.

**Figure 7:**
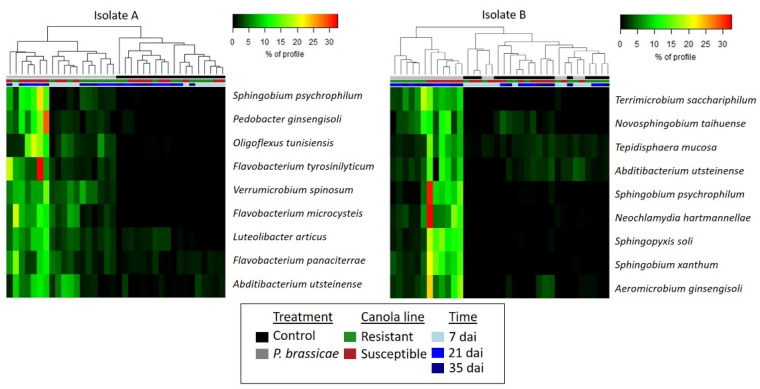
Heatmap depicting bacterial species exhibiting differential abundance in rhizosphere samples. Three replicates of clubroot-resistant and -susceptible canola were sampled at 7, 21, and 35 days after inoculation (dai) with two isolates (A and B) of *Plasmodiophora brassicae* (pathotype 3A). Non-inoculated plants served as controls. Bacterial taxonomy and abundance were obtained from the sequence analysis of the 16S rRNA region using QIIME2. Several bacterial species associated with the rhizosphere were more abundant in inoculated samples, but their abundance differed across canola lines, sampling times, and *P. brassicae* isolates.

Differential abundance analysis was performed to detect the response of bacterial communities associated with the rhizosphere and roots to inoculation. This analysis revealed that the rhizosphere soil of susceptible canola plants inoculated with either isolate exhibited a higher abundance (W > 1000) of bacterial species from the genera *Abditibacterium* and *Sphingobium* (Figure 7). In addition, *Abditibacterium*, *Flavobacterium*, *Luteolibacter*, *Oligoflexus*, *Pedobacter*, *Sphingobium*, and *Verrucomicrobium* were more abundant in the rhizosphere of susceptible plants inoculated with isolate A (Figure 7). Bacterial profiles at the genus level confirmed that the relative abundance of *Flavobacterium* was higher in the rhizosphere of susceptible canola inoculated with isolate A at 21 and 35 dai, compared to resistant inoculated canola and susceptible inoculated canola at 7 dai (Figure 6). In contrast, the genera *Aeromicrobium*, *Neochlamydia*, *Sphingopyxis*, and *Tepidisphaera* were predominant only in the rhizosphere of susceptible canola inoculated with isolate B (Figure 7). The genera *Novosphingobium* and *Terrimicrobium* were more abundant in susceptible and resistant canola samples inoculated with isolate B. At 21 and 35 dai, an increase in the relative abundance of these bacterial species associated with the rhizosphere was observed in response to inoculation with either isolate of *P. brassicae*.

**Figure 8:**
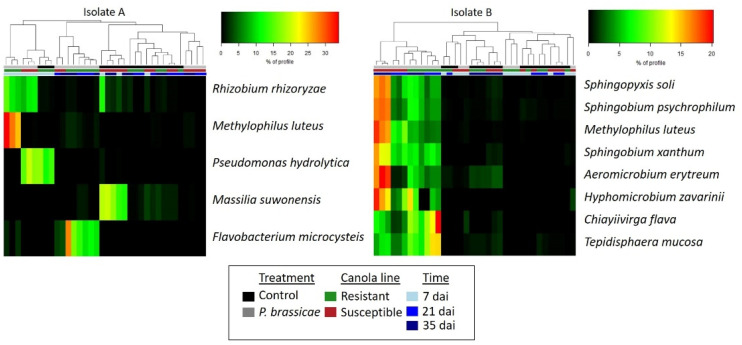
Heatmap depicting bacterial species exhibiting differential abundance in root samples. Three replicates of clubroot-resistant and -susceptible canola were sampled at 7, 21, and 35 days after inoculation (dai) with two isolates (A and B) of *Plasmodiophora brassicae* (pathotype 3A). Non-inoculated plants served as controls. Bacterial taxonomy and abundance were obtained from the sequence analysis of the 16S rRNA region using QIIME2. Several root endophytic bacteria were more abundant in inoculated samples, but their abundance differed across canola lines, sampling times, and *P. brassicae* isolates.

Differential abundance analysis of bacterial communities associated with the root interior of canola identified *Methylophilus luteus* as highly abundant (W > 200) in resistant canola collected at 7 dai with isolate A, as well as in susceptible canola collected at 21 dai or 35 dai with isolate B (Figure 8). Canola roots inoculated with isolate A also exhibited a higher abundance of *Pseudomonas* and *Rhizobium* at 7 dai (Figure 8). *Rhizobium* was predominant in both canola lines, while *Pseudomonas* was abundant in susceptible inoculated plants and resistant uninoculated canola (Figure 6 and Figure 8). *Flavobacterium* was predominant in inoculated resistant plants collected at 21 and 35 dai (Figure 8). Additionally, *Massilia* was more abundant only in control samples of susceptible canola at 21 and 35 dai. In plants inoculated with isolate B, the genera *Aeromicrobium*, *Hyphomicrobium*, *Sphingobium*, and *Sphingopyxis* exhibited a higher abundance in susceptible canola collected at 21 and 35 dai, whereas *Chiayiivirga* and *Tepidisphaera* were more abundant in resistant plants collected at the same time.

## 3. Discussion

The use of resistant canola cultivars is the most effective strategy for managing clubroot in western Canada [4,11], but the emergence of resistant-breaking pathotypes of *P. brassicae* is a serious concern for canola production [5,13]. The pathogen isolates included in our study were classified as pathotype 3A (Figure S5) on the CCD set, which has been identified as the most widespread resistance-breaking pathotype in western Canada [5]. As expected, inoculation with either isolate caused severe clubroot symptoms and high DSI values for the susceptible canola plants (Figure 1). However, resistant canola inoculated with isolate A exhibited higher DSI values compared to those inoculated with isolate B (Figure 1). Previous studies concluded that repeated exposure of a *P. brassicae* population to a particular resistance source can result in a loss in the effectiveness of that resistance, because virulent components of the pathogen population are more likely to reproduce [39,40,41]. *P. brassicae* isolates A and B, designated as pathotype 3A on the CCD set, were selected from a field population of the pathogen collected in 2014. However, isolate A had been utilized in several greenhouse trials prior to our experiment, whereas isolate B was directly derived from the original source. Hence, continuous exposure of isolate A to resistant canola may have led to the selection of more virulent spores, resulting in increased aggressiveness.

Metabarcoding analysis and taxonomic classification at the genus level indicated that the fungi associated with the rhizosphere and root interior, as well as the bacterial communities associated with the roots of canola plants, differed mainly in sampling times (7, 21, and 35 dai) (Figure 2 and Figure 5). Previous studies investigating the impact of plant growth stage on microbial communities associated with the rhizosphere and roots of canola have also noted shifts in fungal and/or bacterial communities across different growth stages [42,43,44,45,46,47]. Another study concluded that changes in microbial diversity across growth stages could be explained by morphological and physiological changes in the root system occurring during plant growth [48]. For example, as plants grow and mature, the increase in root volume and surface area improves the availability of habitats and resources for microbial colonization. Additionally, signaling pathways established between host plants and microorganisms within plant tissues at earlier stages may modulate further colonization at later stages. Moreover, mature plant roots release complex metabolites that can influence microbial communities. Previous studies have also shown that the presence of certain microbial groups at specific plant growth stages is influenced by nutrient availability and microbial ecological strategies [49,50,51,52]. Our results confirm the abundance of Proteobacteria genera, such as *Acinetobacter*, *Pantoea*, *Pedobacter*, and *Pseudomonas*, in roots collected at 7 dai, suggesting efficient utilization of carbon sources provided by root exudates during the early colonization of canola roots (r-strategy) (Figure 6). Conversely, the higher abundance of Actinobacteria, such as *Streptomyces* at 21 and 35 dai (Figure 6), may be associated with a low growth rate and tolerance for disturbance (K-strategy), enabling their persistence at later stages of canola growth. However, at 21 and 35 dai, a high abundance of *Buchnera* (Proteobacteria) and *Flavobacterium* (Bacteroidetes) was also detected, whereas Actinobacteria such as *Arthrobacter* and *Mycobacterium* were predominant in certain samples collected at an early stage (7 dai) (Figure 6). These results indicate that not all members of microbial communities fit neatly into r- or K-strategist categories [53]. Nevertheless, employing classification based on ecological strategies can still provide valuable insights into the dynamics of microbial communities [52]. 

Broad fungal taxonomic groups can also be classified as r-strategists or K-strategists based on their ecological characteristics. Generally, most copiotrophic fungi, such as Ascomycota and Zygomycota, are classified as r-strategists, whereas oligotrophic fungi and ectomycorrhizal fungi such as Basidiomycota are K-strategists [53,54]. Our results show that at an early plant stage (7 dai), the abundance of *Fusarium* associated with canola roots was higher for samples inoculated with *P. brassicae* isolate B (Figure 3). The genus *Fusarium,* belonging to the Ascomycota, includes several species described as r-strategists. During their saprotrophic phase, these species exhibit rapid growth when fresh organic matter is available [55]. This accelerated growth can be attributed to the production of enzymes involved in the decomposition of organic matter [56]. The genera *Candida* and *Phialemonium*, also classified as Ascomycota, exhibited a higher abundance in samples collected at 7 and 21 dai (Figure 3). Both genera include several species known for their ability to grow on a wide variety of substrates [57,58], which allows their fast growth at early plant stages. In contrast, the genus *Olpidium* was highly abundant at 21 and 35 dai, emerging as the predominant fungus in the root samples inoculated with isolate B during those time points (Figure 3). Additionally, *Olpidium* was also predominant in rhizosphere soil samples collected 35 dai with isolate B (Figure 3). This genus was predominantly represented by *O. brassicae*, an obligate parasite of root epidermal cells, particularly in the Brassicaceae [59]. Similar to *P. brassicae*, the fungus *O. brassicae* forms resting spores capable of surviving for many years in the soil. Infection of host tissues by *O. brassicae* involves several stages, including primary and secondary infections [60]. These sequential steps during the root colonization process may explain why this fungus appeared to be more abundant at later time points. 

Microbial profiles revealed changes in the fungal communities associated with the rhizosphere of both canola lines upon inoculation with either *P. brassicae* isolate. For example, the profiles of rhizosphere fungal communities differed between controls and samples inoculated with isolate B at 35 dai, while samples inoculated with isolate A exhibited differences only across sampling times (Figure 2). Permanova results also indicate a significant interaction of inoculation and time for isolate B, whereas for isolate A, only a significant effect of sampling time was detected, suggesting distinct effects for both isolates (Table 1). For the root samples, analysis of fungal and bacterial profiles showed a significant effect of *P. brassicae* isolate and inoculation treatment (Figure 2 and Figure 5, Table 1). Bacterial profiles indicated that even at an early stage (7 dai), the bacterial communities associated with resistant canola inoculated with isolate A differed from those of most samples collected at the same time. In contrast, for isolate B, most samples showed a similar community composition (Figure 5). The bacterial profiles of most control samples collected at 21 and 35 dai were similar. However, for both *P. brassicae* isolates, bacterial communities associated with resistant inoculated samples at 21 dai differed from those of other samples. This indicates that inoculation induced changes in bacterial communities in the resistant canola line at 21 dai, regardless of the isolate type (Figure 5). Permanova results show significant interactions of sampling time and inoculation, as well as canola line and inoculation, in the bacterial communities associated with roots for both isolates (Table 1). The results with isolate B suggest that inoculation with *P. brassicae* can induce changes in the bacterial and fungal communities associated with the rhizosphere and roots. Other studies investigating the effect of clubroot on the microbiome of *Brassica* plants found differences between asymptomatic roots and symptomatic roots in the bacterial and fungal populations associated with *B. napus* [35] and in the bacterial populations associated with *B. juncea* [21]. Similarly, differences in the bacterial microbiome between highly *P. brassicae*-infested and healthy soils sown with Chinese cabbage [61] and cauliflower [62] have been reported. Clubroot development results in extreme modifications of the host root system, which reduces the capacity of plants for water and nutrient uptake [13]. During the formation of root galls, the pathogen alters host metabolism, especially plant hormones such as auxins and cytokinins, as well as other hormones that act as signaling molecules to prevent drought stress or are involved in defense regulation [63]. Previous studies indicated that modifications to plant metabolism in response to *P. brassicae* infection result in changes to the composition of root exudates [64,65,66]. Therefore, a diseased plant may attract and interact with different types of microorganisms due to the production of different metabolites [67]. Inoculation with a highly virulent isolate of *P. brassicae* can cause more severe modifications of the root system and host plant metabolism, leading to more pronounced changes in the rhizosphere and root microbiomes. 

Classification of the fungal communities associated with the rhizosphere and roots of canola lines at the genus level indicated a significant shift in the relative abundance of some genera in response to *P. brassicae* isolate. For example, in rhizosphere samples corresponding to isolate A, *Candida* was highly abundant, whereas for isolate B, the genera *Olpidium* and *Trichoderma* were also abundant (Figure 3). Similarly, in the roots, the abundance of *Arthrobotrys* was higher in samples corresponding to isolate A, whereas *Mortierella* was predominant in samples corresponding to isolate B (Figure 3). These changes in fungal profiles between samples corresponding to different *P. brassicae* isolates (A and B) suggest that the virulence of the isolates influences fungal composition in the rhizosphere and roots. For both isolates, the genus *Mortierella* was predominant in the rhizosphere, while *Fusarium* and *Olpidium* were predominant in the root interior (Figure 3). *Mortierella* is a known saprophyte that is well represented in the rhizosphere and roots of canola [68] and has been previously detected in the Canadian prairies [69,70]. The genus *Fusarium* includes several pathogenic species, such as *F. acuminatum*, *F. avenaceum*, *F. culmorum*, *F. equiseti*, *F. graminearum*, *F. oxysporum*, *F. solani*, *F. sporotrichioides*, and *F. torulosam*, which cause various canola diseases, including crown rot, seedling blight, root rot, and Fusarium wilt [70,71,72]. *Fusarium* was previously reported in the rhizosphere microbiome of canola cultivated in the Canadian prairies [69,73,74]. As noted earlier, *Olpidium* is a pathogenic fungus associated with the Brassicaceae and was reported to be the predominant genus in the rhizosphere and roots of canola grown in western Canada [69,70,73,74]. Differential abundance analysis of fungal communities indicated that the relative abundance of *O. brassicae* increased in the root interior of samples inoculated with either *P. brassicae* isolate at 21 and 35 dai (Figure 4). Similarly, *F. solani* was more abundant in the rhizosphere at 35 and 21 dai with isolates A and B, respectively (Figure 4), whereas in the root, this genus was more abundant in inoculated samples collected at 7 and 35 dai for both isolates (Figure 4). Collectively, these results suggest that infection by *P. brassicae* may result in increased susceptibility to infection or colonization by other pathogens such as *O. brassicae* or *F. solani*.

Bacteria associated with the root endosphere varied greatly between canola lines and isolates, whereas the relative abundance of bacterial communities in the rhizosphere tended to be more similar (Figure 5 and Figure 6). These results suggest that the root endophytic microbiome was more sensitive to factors such as canola genotype or inoculation. Previous studies demonstrated that plant species and/or cultivar, plant growth stage, and plant health can influence interactions between a host crop and bacterial endophytes [36]. Additional factors mediating this interaction include differences in root morphology, composition of root exudates [33], and the selection of particular endophytic microbiota by the plant immune system [75]. Alterations in the root system and the activation of defense mechanisms in response to *P. brassicae* infection in susceptible canola lines may also influence the composition of endophytic bacterial communities. Several bacterial genera that were predominant in the rhizosphere and/or root samples, such as *Arthrobacter*, *Bacillus*, *Massilia*, *Methylophilus*, *Pseudomonas*, *Pantoea*, *Pedobacter*, *Pseudomonas*, *Rhizobium*, *Sphingomonas*, and *Streptomyces* (Figure 6), were previously reported in the rhizosphere and/or root interior microbiomes associated with canola [73,74,76,77,78].

Differential abundance analysis detected several bacterial species associated with the rhizosphere and roots that were more abundant in inoculated samples (Figure 7 and Figure 8). However, their abundance differed across canola lines, sampling times, and *P. brassicae* isolates. Most of the bacteria exhibiting a higher abundance in inoculated treatments were found in the susceptible canola line (Figure 7 and Figure 8). Additionally, an increase in the relative abundance of bacterial species associated with susceptible canola was detected at 21 and 35 dai with either isolate in most of the samples, except for *Pseudomonas*. The latter was more abundant in susceptible plants inoculated with isolate A and collected at 7 dai (Figure 8). In contrast, a lower number of bacterial genera, such as *Chiayiivirga*, *Flavobacterium*, and *Tepidisphaera*, exhibited a higher abundance in resistant inoculated canola, while some species such as *Methylophilus*, *Novosphingobium*, *Rhizobium*, and *Terrimicrobium* were abundant in inoculated plants of both canola lines (Figure 7 and Figure 8). The greater abundance of *Flavobacterium* and other bacteria in resistant canola may be associated with the selection of a specific bacterial microbiome by resistant plants in response to *P. brassicae* infection. This suggests that these bacteria may have potential antagonistic properties that can contribute to clubroot control. A previous study comparing the microbiome of clubroot-symptomatic and -asymptomatic *B. napus* plants concluded that many bacteria were unique to the asymptomatic samples and that many may be beneficial for the plant [35]. Some genera that were predominant in resistant canola, such as *Flavobacterium*, *Methylophilus*, *Novosphingobium*, and *Rhizobium*, were previously detected in the rhizosphere and roots of canola [44,73,77,79]. Additionally, some bacteria identified in our study were previously shown to be plant growth promoters that can enhance canola growth and/or health. For instance, *Flavobacterium* spp. were capable of stimulating root elongation of *B. juncea* seedlings either in the presence or absence of toxic Cd concentrations by producing indoles, siderophores, and the ACC deaminase enzyme [80]. *Flavobacterium* spp. seed endophytes are phosphate solubilizers and indole-3-acetic acid producers with a high tolerance to salinity and osmotic stress in rice [81]. *Methylophilus* is a methylotrophic bacterium capable of mitigating different types of biotic and abiotic stress [82]. *Novosphingobium* spp. produce siderophores and can stimulate the growth of canola and rice [44,83]. Several *Rhizobium* strains have been reported to promote plant growth of *B. napus* under salinity stress [84]. In addition, the inoculation of canola seeds with certain strains of *Rhizobium leguminosarum* was found to increase early seedling root growth under gnotobiotic conditions [85]. 

Previous studies identified several bacterial species as potential biocontrol agents for clubroot. For example, *Bacillus* spp., including *B. amyloliquefaciens*, *B. subtilis*, and *B. velezensis*, have been shown to inhibit *P. brassicae* infection and reduce clubroot severity in greenhouse trials [22,23,26]. These bacteria can produce hydrolytic enzymes and diverse secondary metabolites with antagonistic properties [30,31]. Some species of *Streptomyces*, such as *S. alfalfae* and *S. platensis*, have also shown antagonistic effects against clubroot [86,87]. The inoculation of *B. juncea* with *Zhihengliuella aestuarii* induced defense mechanisms against *P. brassicae* infection [24]. In addition, culture filtrates of several strains of *Lysobacter antibioticus* isolated from rhizosphere soil reduced clubroot severity in Chinese cabbage [25]. In our study, the relative abundance of the abovementioned genera in the rhizosphere and root did not show significant variation in response to *P. brassicae* inoculation of canola, as determined by the differential abundance analysis (Figure 7 and Figure 8). While many bacterial species have been previously described as plant promoters and/or biocontrol agents, our approach to mitigating clubroot considers the application of a microbial consortium based on the shifts in root-associated microbial profiles in response to *P. brassicae* inoculation. The application of a mixed community of microorganisms for disease management can improve the efficacy of disease-inhibiting microbes and emulate native conditions that occur in a natural soil–root system [61]. A recent study using a community of bacteria, including *Bacillus cereus*, *Lysobacter antibioticus*, and *Lysobacter capsica*, reduced clubroot incidence and showed a strong biocontrol effect on Chinese cabbage [27], highlighting the potential of microbial consortia as a tool to manage this and other soilborne diseases. Additional experiments are underway to generate more precise information on the microbial profiles associated with canola and to expand the spectrum of biocontrol candidates. 

## 4. Materials and Methods

### 4.1. Plant and Pathogen Material 

This study included clubroot-resistant (12PH0277) and -susceptible (12PH0244) doubled-haploid lines of *B. napus*, which were derived from F_1_ plants of the cross 11SR0099 (clubroot-resistant) × 12DH0001 (clubroot-susceptible). The canola cv. ‘Westar’ was also included in clubroot disease assessments as a universally susceptible check to ensure the viability of pathogen inoculum and conditions conducive to disease development. Two field isolates of *P. brassicae*, designated as isolate A and isolate B and classified as pathotype 3A on the CCD set [5], were used to inoculate the hosts. Both *P. brassicae* isolates were selected from a field population of the pathogen collected from canola fields in Sturgeon County, Alberta, Canada, in 2014. However, isolate A had been utilized in several greenhouse trials prior to our experiment, whereas isolate B was directly derived from the original source. The galls from canola plants containing the *P. brassicae* isolates were stored at −80 °C prior to use in the inoculation experiments. Inoculum was prepared by macerating root galls using a variable speed blender at high speed for 3 min followed by blending twice for 2 min each time, filtering the spore suspension through three layers of cheesecloth, and adjusting the spore concentration to 1 × 10^7^ resting spores mL^−1^ [86].

### 4.2. Inoculation, Greenhouse Conditions, and Experimental Design

A Black Chernozemic soil [88] was collected on 6 August 2021 from an organic field located on the South Campus of the University of Alberta, Edmonton, Canada. This field had no previous history of clubroot. The soil was sampled at around 15 cm in depth, air dried, and passed through a 4 mm sieve. The sampled soil was mixed with Sunshine LA4 potting mixture (Sunshine Growers, Vancouver, BC, Canada) at a 2:1 (*v*:*v*) ratio in a soil mixer for 1 h. Canola seeds were germinated on moistened filter paper (moistened with 1.5 mL of autoclaved distilled water) in Petri dishes for 1 week prior to inoculation. The root system of each canola seedling was dipped in a *P. brassicae* resting spore suspension (1 × 10^7^ resting spores mL^−1^) for 15 s before being transferred to Cone-tainers (length = 21 cm, diam. = 3.8 cm; Stuewe and Sons., Inc., Tangent, OR, USA) filled with 100 g of soil mixture [86]. Seedlings were planted at a density of one plant per Cone-tainer, with an additional 1 mL of spore suspension (1 × 10^7^ resting spores mL^−1^) added directly onto the soil around each plant after transplanting to ensure strong disease pressure. The Cone-tainers were placed in a greenhouse maintained at 22 °C with a 16 h photoperiod under natural light supplemented by artificial lighting. The soil mix was saturated with water (pH~6.5) for the first week following inoculation. Subsequently, seedlings were watered and fertilized with a 20 N:20 P:20 K solution as needed. Control treatments did not receive any inoculum. 

Three biological replicates, each consisting of 24 plants, were included for each line and treatment (inoculated and non-inoculated). Samples were harvested at 7, 21, and 35 dai to determine microbial diversity and abundance. An additional three replicates per treatment were maintained for a final assessment of clubroot disease severity at 42 dai, as described below. Two similar experiments were conducted, changing only the *P. brassicae* isolate (A and B) between them. Individual replicates were arranged in a completely randomized design and rotated on each harvest day (Figure S6).

### 4.3. Sample Collection

At each harvest time, 72 Cone-tainers were randomly selected for each line and treatment. Plants with attached soil were carefully extracted from the pots using a spatula. The shoots were separated from the roots, and soil loosely attached to the roots was removed by gently shaking them. Plants with adhering rhizosphere soil from the same replicate were pooled together in a labelled plastic bag and kept at 4 °C until processing.

Rhizosphere soil was separated from the roots by transferring to a flask filled with a sterile phosphate-buffer saline (PBS) diluent (NaCl, KH_2_PO_4_, Na_2_HPO_4_, MgSO_4_, gelatin) based on a 1:100 ratio of root weight (g) to solution (mL) [43,89]. The roots were shaken on a rotary shaker (200 rpm) for 20 min [43]. The resulting soil/PBS solution was centrifuged for 10 min at 8000× *g* to collect the rhizosphere soil while the supernatant was discarded [89]. The collected rhizosphere soil was stored at −80 °C for further molecular analyses. Roots were washed with sterile tap water and surface-disinfested by transferring them to a flask filled with NaClO (1.05% *v*/*v*) in sterile diluent using a 1:40 ratio of root weight (g) to solution (mL) [90]. The roots were shaken on a rotary shaker (200 rpm) for 10 min. Subsequently, they were rinsed four times with sterile diluent to remove disinfectant solution. Finally, the roots were frozen using liquid nitrogen and stored at −80 °C for molecular analyses. 

### 4.4. DNA Extraction and Microbiome Profiling 

Total genomic DNA was extracted from the rhizosphere soil and roots using a DNeasy PowerSoil Pro Kit or DNeasy Plant Pro Kit (Qiagen Inc., Toronto, ON, Canada), respectively. Root samples (~100 mg) were ground to a powder using a mortar and pestle after adding liquid nitrogen. Rhizosphere soil samples (~250 mg) were homogenized with a lysis solution in Qiagen PowerBead tubes using a Vortex-Genie2 (Scientific Industries, Bohemia, NY, USA) at 2700 rpm for 10 min. The remaining DNA extraction steps were conducted following the manufacturer’s protocols. The yield was measured using a Qubit DNA HS Assay Kit (Thermo Fisher Scientific, Waltham, MA, USA). Aliquots of the DNA were visualized by gel electrophoresis in 1% agarose stained with SYBR safe DNA stain (Invitrogen, Carlsbad, CA, USA) and compared with a DNA mass ladder (Invitrogen) using a Bio-Rad Gel Doc XR System (Bio-Rad Laboratories, Mississauga, ON, Canada). Total DNA extracted from the rhizosphere and root endophytic communities was submitted to Omega Bioservices (Norcross, GA, USA) for sequencing of the 16S rRNA (bacteria) and ITS (fungi) regions using an Illumina MiSeq platform (2 × 250 bp paired ends). The barcode primers used for amplification of the 16S rRNA region included Bakt_341F (CCTACGGGNGGCWGCAG) and Bakt_805R (GACTACHVGGGTATCTAATCC) [91], while the primers used for the amplification of the ITS region consisted of ITS1F (CTTGGTCATTTAGAGGAAGTAA) and ITS4 (TCCTCCGCTTATTGATATGC) [92].

### 4.5. Bioinformatics and Statistic Analyses 

Microbiome bioinformatics were conducted using QIIME2 2022.8 [93] with slight modifications based on the official tutorials (https://docs.qiime2.org/2022.8/tutorials/ (accessed on 3 February 2022)). Sequences derived from bacterial and fungal communities were processed separately for both *P. brassicae* isolates. The raw paired end reads were treated with Cutadapt to remove Illumina adaptors, followed by denoising with DADA2 (Divisive Amplicon Denoising Algorithm). Moreover, the QIIME2 taxa filter-table plugin was used to remove all features annotated as mitochondria and chloroplast from the bacterial profiles. In the rhizosphere, the percentage of reads not assigned to bacteria was 2.6% and 3.5% for isolate A and B, respectively, whereas in the roots, it was 63.2% and 68.7% for isolate A and B, respectively. The percentage of reads not assigned to fungi in the rhizosphere was 33.1% and 25.3% for isolate A and B, respectively, whereas in the roots, it was 57.7% and 52.4% for isolate A and B, respectively. Taxonomic information for each ASV was assigned using pre-trained Naive Bayes classifiers for bacteria (SILVA v.138) and fungi (UNITE ver8_99_04.02.2020). Phylogenetic analysis was conducted using the QIIME2 align-to-tree-mafft-fasttree plugin to generate a multiple sequence alignment of sequences, followed by the maximum-likelihood procedure implemented in Fasttree to infer the phylogenetic tree. 

The principal coordinates analysis (PCoA) describing the diversity between the samples (β-diversity) based on Bray Curtis dissimilarity metrics was inferred by applying the q2-diversity plugin. The statistical significance of the clustering pattern was evaluated by using Permutational Multivariate Analysis of Variance (PERMANOVA). The differential abundance analysis of the observed ASVs among treatments was conducted by ANCOM (Analysis of Composition of Microbiomes), which relies on a compositional-aware approach allowing identification of differentially abundant taxa. Before ANCOM, the low abundant ASVs (<5 reads or <10% of samples) were removed using the filter-features plugin to improve the inference of ASVs that are differentially abundant. The W statistic is the number of ANCOM sub-hypotheses that have passed for each individual ASV, indicating that the ratios of that ASV’s relative abundance to the relative abundances of other taxa were significantly different. Heatmaps showing the differentially abundant ASVs between samples were generated using the VEGAN package (version 2.6-4) in R Studio (version 2022.02.3 Build 492) [94]. The sequence data can be accessed in NCBI with the accession number PRJNA1044771.

### 4.6. Disease Assessment 

Three independent biological replicates, each consisting of 24 plants of each doubled-haploid *B. napus* line alongside the susceptible check cultivar ‘Westar’, were rated for clubroot development in all experiments [95]. Disease rating was performed at 42 dai following a 0–3 scale [96]. The individual ratings were used to calculate the disease severity index using the following formula: DSI (%) = [(n1 × 1 + n2 × 2 + n3 × 3)/(n × 3)] × 100, where n1, n2, and n3 refer to the number of plants in each symptom severity class and n refers to the total number of plants tested [1].

### 4.7. Pathotype Composition 

The pathotype classification of *P. brassicae isolates* A and B was assessed on the 13 differential hosts of the Canadian Clubroot Differential set [5]. The experiment was arranged in a completely randomized design with four replicates per host line and 12 seedlings per experimental unit. Plant inoculations and growth conditions were as described above. The inoculated seedlings were planted in plastic pots (6 cm × 6 cm × 6 cm) filled with Sunshine LA4 potting mixture (Sunshine Growers, Vancouver, BC, Canada), with one seedling per pot. The plants were maintained for 6 weeks after inoculation, at which time they were assessed for clubroot symptom development as described above.

## Figures and Tables

**Table 1:** Permanova analysis (*p*-values) of the fungi and bacteria community profiles associated with the rhizosphere and roots of clubroot-resistant and clubroot-susceptible canola lines. Three replicates from clubroot-resistant and -susceptible canola lines were sampled at 7, 21, and 35 days after inoculation (dai) with two isolates (A and B) of *Plasmodiophora brassicae* (pathotype 3A). Non-inoculated plants served as controls. Microbial profiles were obtained from the sequence analysis of the 16S rRNA (bacteria) and ITS (fungi) regions using QIIME2. A significant effect of sampling time was detected in the fungal and bacterial communities associated with the rhizosphere for both isolates. Clubroot inoculation, as well as the interaction of clubroot inoculation and sampling time, significantly influenced the bacterial and fungal communities in the roots.

Factors	Fungi				Bacteria			
	Rhizosphere		Root		Rhizosphere		Root	
	Isolate A	Isolate B	Isolate A	Isolate B	Isolate A	Isolate B	Isolate A	Isolate B
Canola line	0.49	0.54	0.36	0.33	0.06	0.22	0.15	0.06
Clubroot inoculation	0.17	0.05	**0.03**	**0.01**	0.08	0.09	**0.0002**	**0.0002**
Sampling time	**0.0002**	**0.0002**	**0.0002**	**0.0002**	**0.0002**	**0.0002**	**0.0002**	**0.0002**
Canola line × Clubroot inoculation	0.36	0.78	0.08	0.2	0.29	0.48	**0.0004**	**0.01**
Canola line × Sampling time	0.37	0.18	0.08	0.06	**0.03**	**0.04**	0.1	0.1
Clubroot inoculation × Sampling time	0.08	**0.006**	**0.001**	**0.0002**	**0.04**	**0.04**	**0.0002**	**0.0002**

Note: *p*-values highlighted in bold denote a significant effect (*p* < 0.05) in bacterial or fungal communities.

## Data Availability

The sequence data analyzed in this study can be accessed in NCBI with the accession number PRJNA1044771.

## Supplementary Materials

The following supporting information can be downloaded at https://www.mdpi.com/article/10.3390/plants13131880/s1. Figure S1: Canola lines 12PH0277 (resistant) and 12PH0244 (susceptible), tested for resistance to clubroot pathotype 3A, harvested at 7, 21, and 35 days after inoculation (isolate A); Figure S2: Canola lines 12PH0277 (resistant) and 12PH0244 (susceptible), tested for resistance to clubroot pathotype 3A, harvested at 7, 21, and 35 days after inoculation (isolate B); Figure S3: Roots and shoots of canola lines 12PH0277 (resistant) and 12PH0244 (susceptible), tested for resistance to clubroot pathotype 3A, harvested at 7, 21, and 35 days after inoculation (isolate A); Figure S4: Roots and shoots of canola lines 12PH0277 (resistant) and 12PH0244 (susceptible), tested for resistance to clubroot pathotype 3A, harvested at 7, 21, and 35 days after inoculation (isolate B); Figure S5: Reaction of the hosts of the Canadian Clubroot Differential Set to inoculation with clubroot; Figure S6: Greenhouse experimental setup.
