# Supplementary figures and images for: Clubroot-Induced Changes in the Root and Rhizosphere Microbiome of Susceptible and Resistant Canola
Source: Plants (Basel). 2024 Jul 8;13(13):1880. doi: 10.3390/plants13131880 (PMC11244039; doi:10.3390/plants13131880)

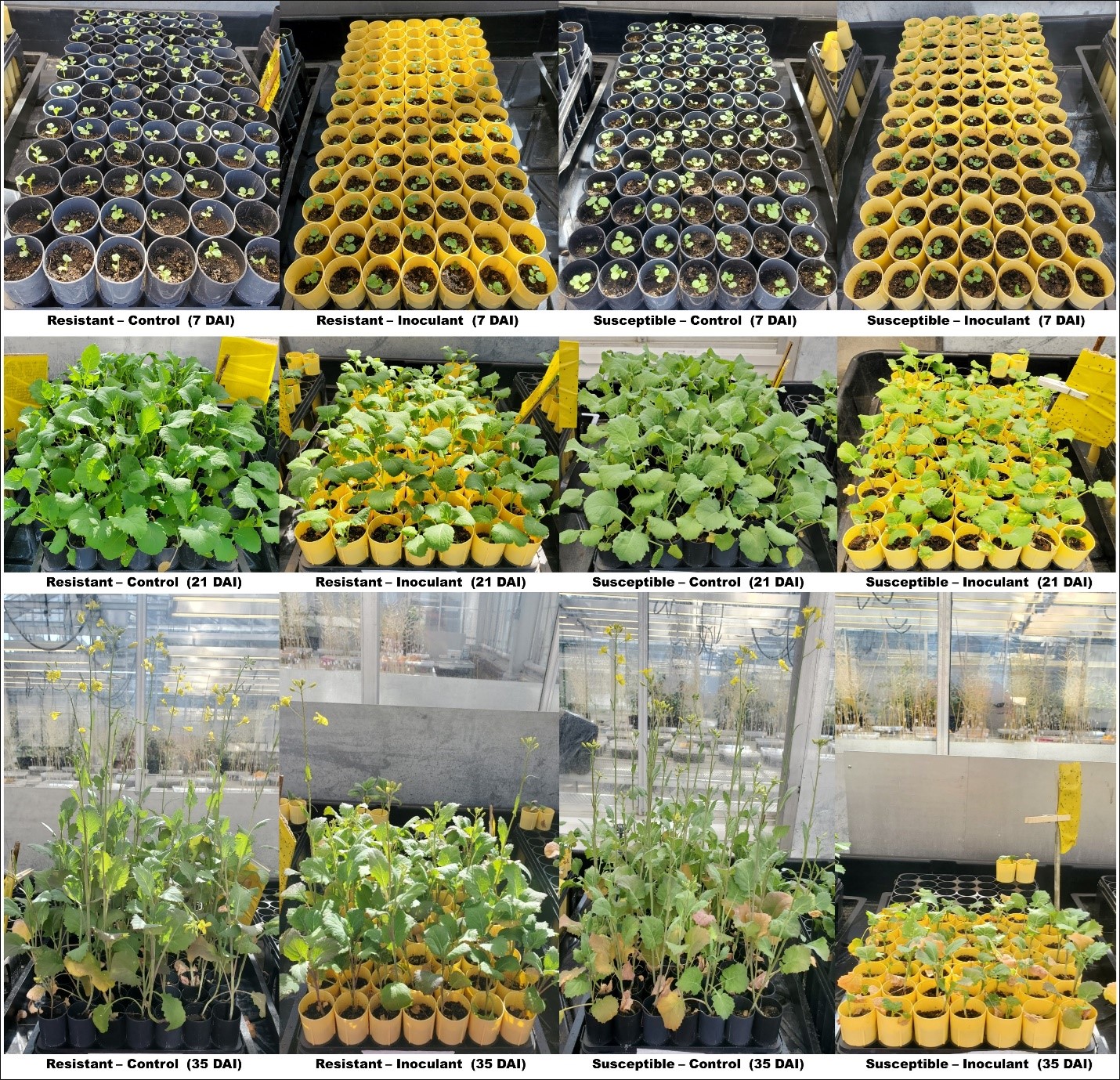

Supplement: Supplementary file 1 [file plants-13-01880-s001.zip › Figure S1.jpg]

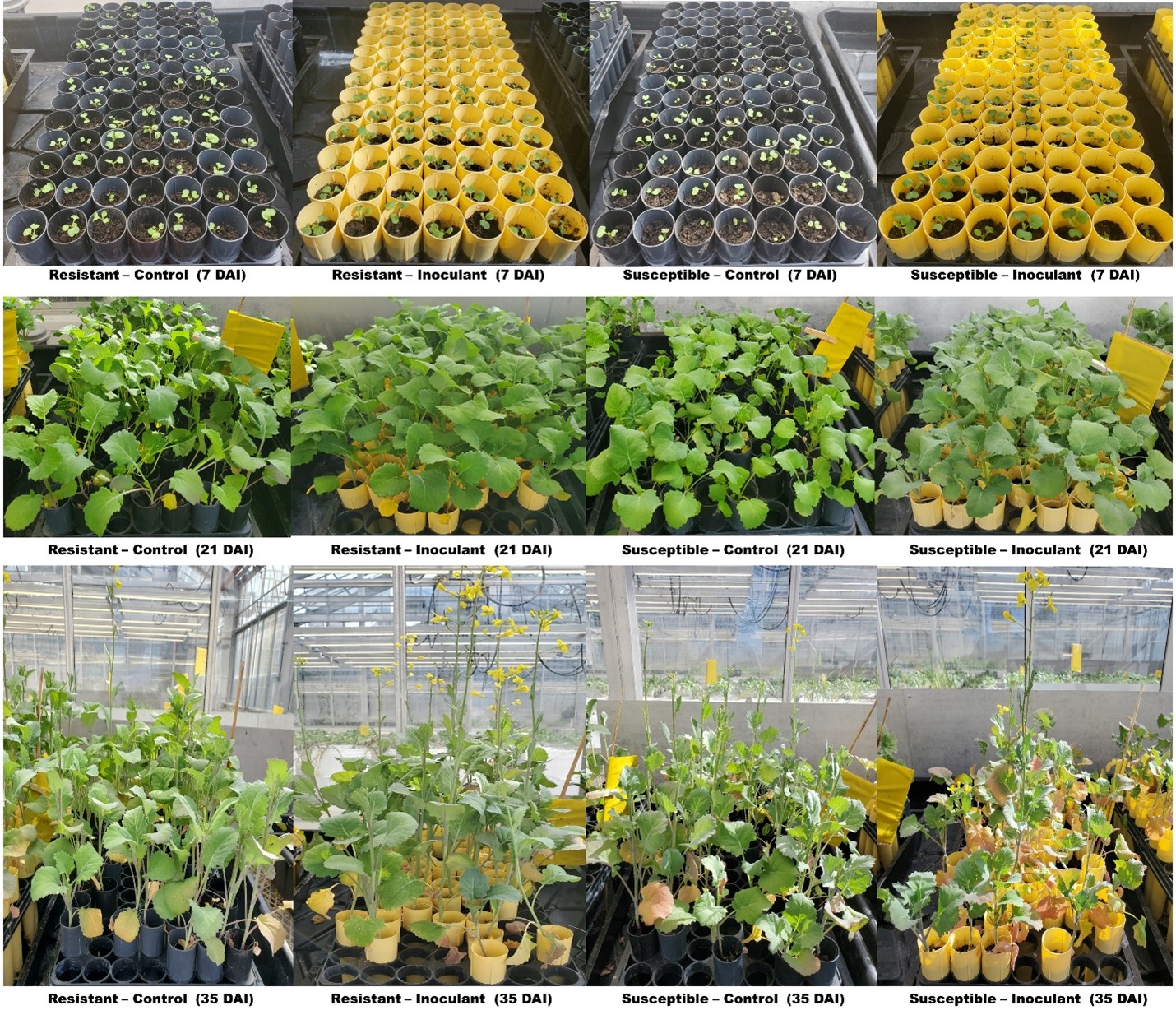

Supplement: Supplementary file 1 [file plants-13-01880-s001.zip › Figure S2.jpg]

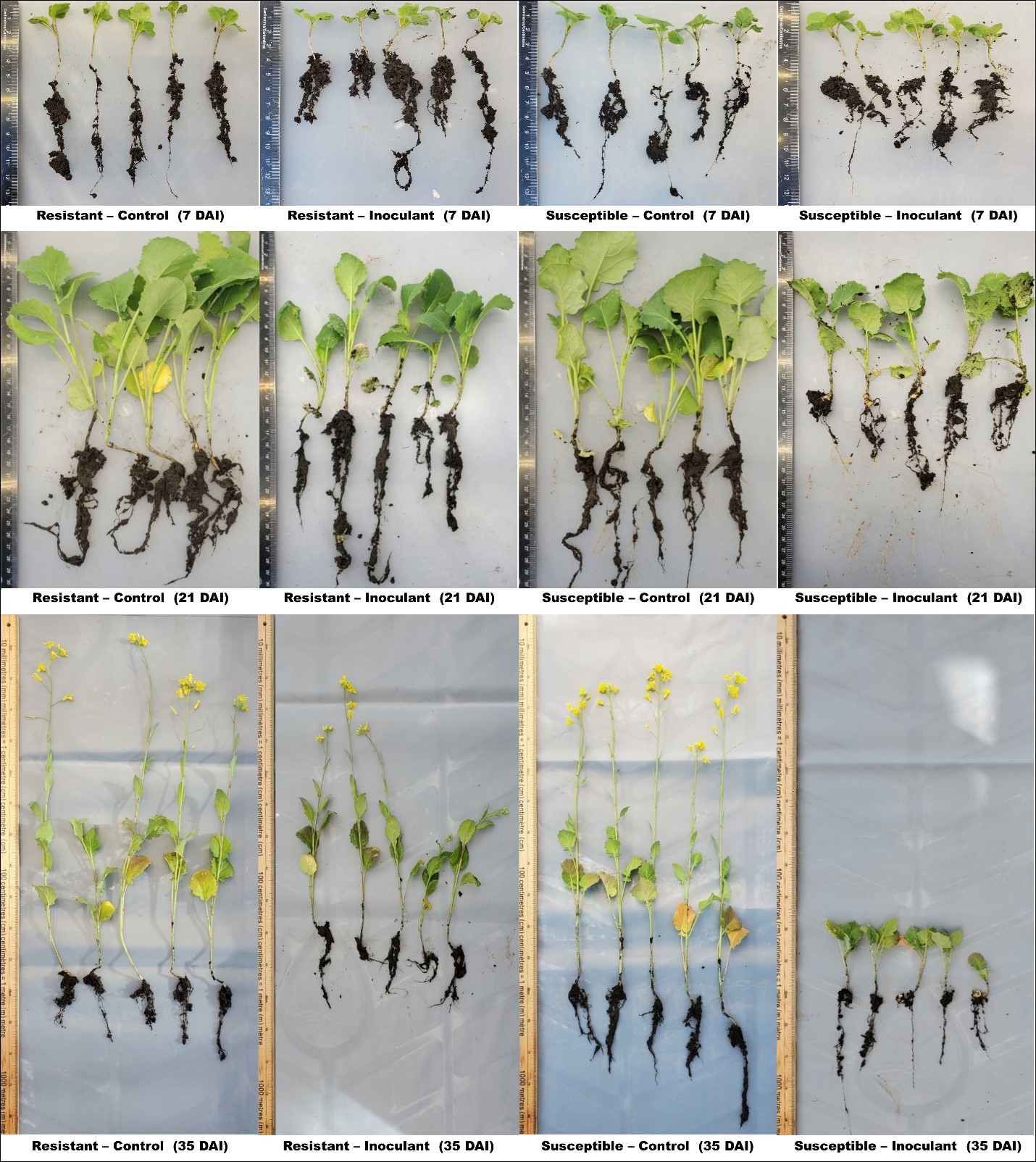

Supplement: Supplementary file 1 [file plants-13-01880-s001.zip › Figure S3.jpg]

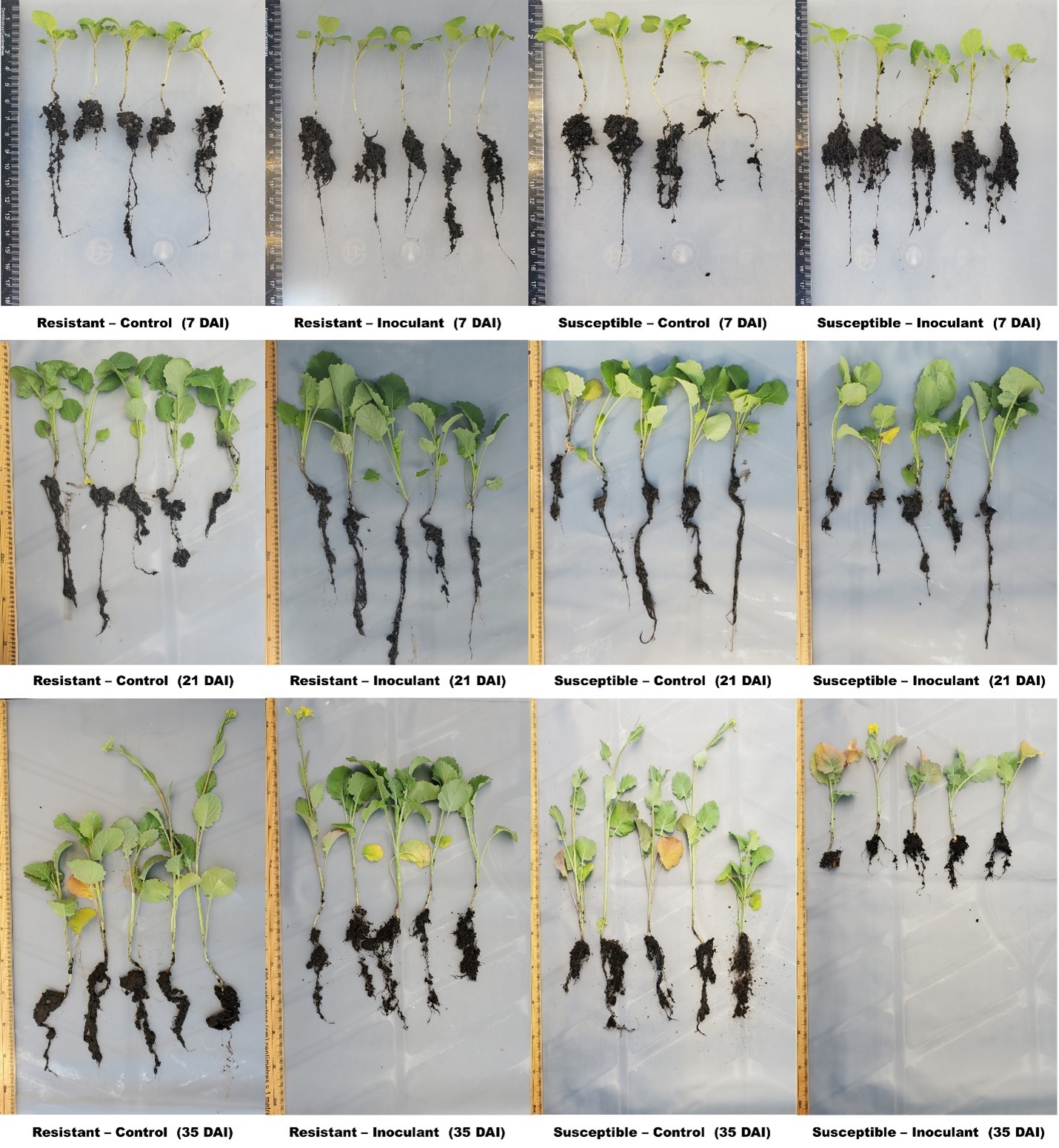

Supplement: Supplementary file 1 [file plants-13-01880-s001.zip › Figure S4.jpg]

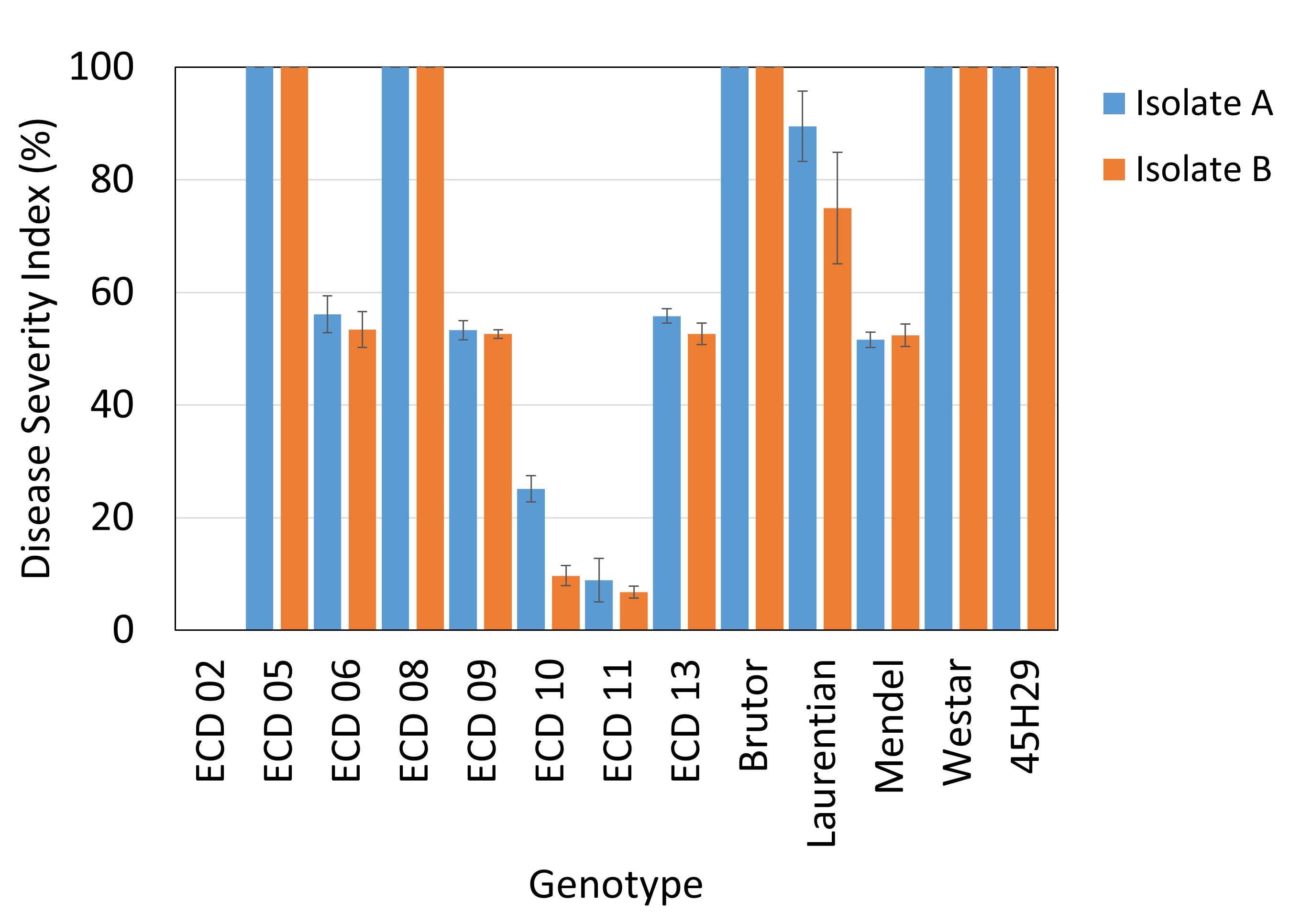

Supplement: Supplementary file 1 [file plants-13-01880-s001.zip › Figure S5.png]

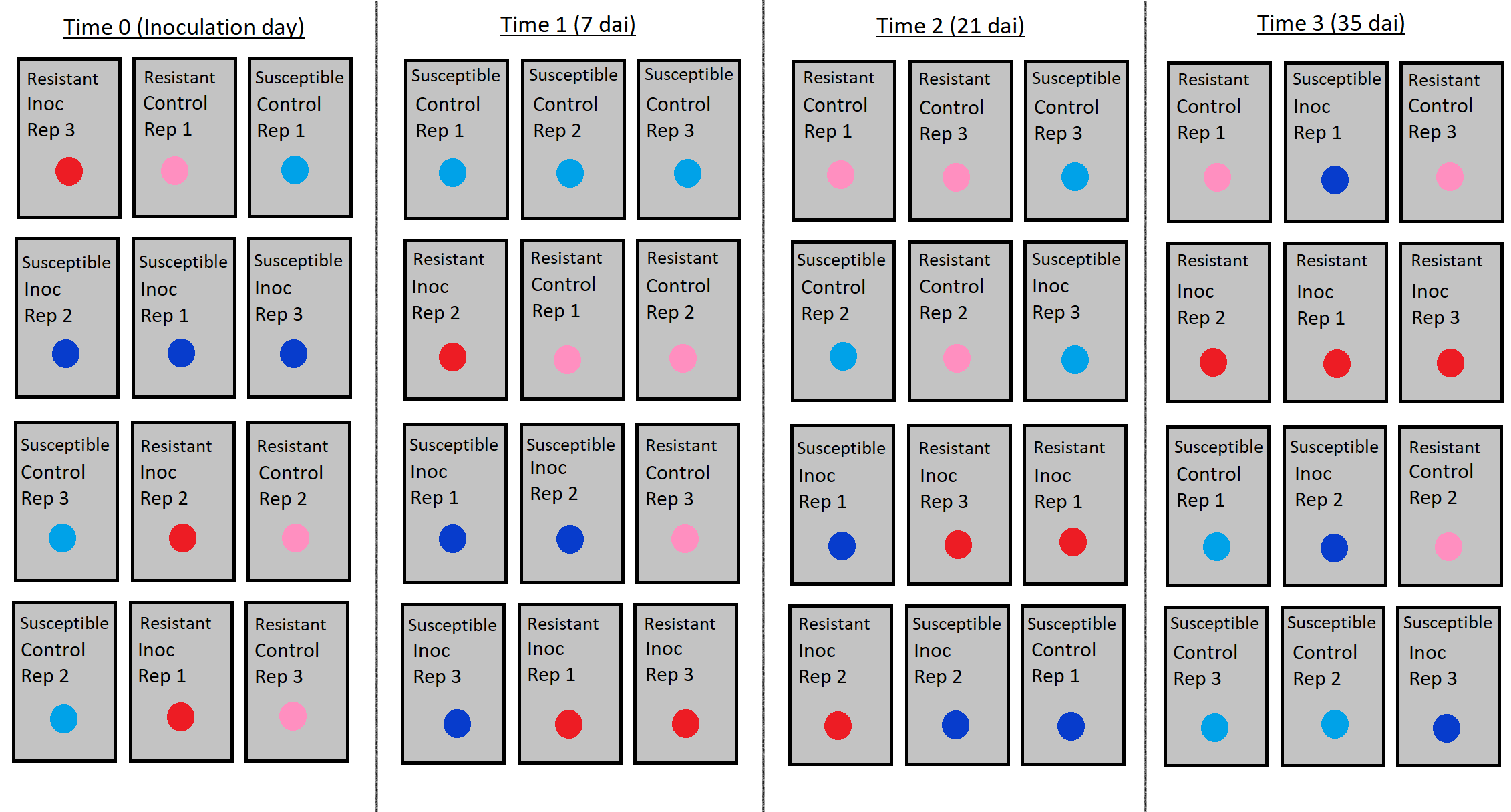

Supplement: Supplementary file 1 [file plants-13-01880-s001.zip › Figure S6.png]
